# Poly-arginine peptides reduce infarct volume in a permanent middle cerebral artery rat stroke model

**DOI:** 10.1186/s12868-016-0253-z

**Published:** 2016-05-03

**Authors:** Diego Milani, Vince W. Clark, Jane L. Cross, Ryan S. Anderton, Neville W. Knuckey, Bruno P. Meloni

**Affiliations:** Centre for Neuromuscular and Neurological Disorders, The University of Western Australia, Nedlands, Australia; Department of Neurosurgery, Sir Charles Gairdner Hospital, QEII Medical Centre, Nedlands, WA Australia; Western Australian Neuroscience Research Institute, A Block, 4th Floor, QEII Medical Centre, Verdun St, Nedlands, WA 6009 Australia; School of Heath Sciences, The University of Notre Dame Australia, Fremantle, WA Australia

**Keywords:** Poly-arginine peptides, Middle cerebral artery occlusion, Stroke, Neuroprotection

## Abstract

**Background:**

We recently reported that poly-arginine peptides have neuroprotective properties both in vitro and in vivo. In cultured cortical neurons exposed to glutamic acid excitotoxicity, we demonstrated that neuroprotective potency increases with polymer length plateauing at R15 to R18 (R = arginine resides). In an in vivo study in rats, we also demonstrated that R9D (R9 peptide synthesised with D-isoform amino acids) administered intravenously at a dose of 1000 nmol/kg 30 min after permanent middle cerebral artery occlusion (MCAO) reduces infarct volume. Based on these positive in vitro and in vivo findings, we decided to examine the neuroprotective efficacy of the L-isoform poly-arginine peptides, R12, R15 and R18 when administered at a dose of 1000 nmol/kg 30 min after permanent MCAO in the rat.

**Results:**

At 24 h post-MCAO, there was reduced total infarct volume for R12 (12.8 % reduction) and R18 (20.5 % reduction), but this reduction only reached statistical significance for R18. Brain slice analysis revealed significantly reduced injury in coronal slices 4 and 5 for R18, and slice 5 for R12. The R15 peptide had no effect on infarct volume. Peptide treatment did not reveal any statistical significant improvement in functional outcomes.

**Conclusion:**

While these findings confirm the in vivo neuroprotective properties of poly-arginine peptides, additional dose studies are required particularly in less severe transient MCAO models so as to further assess the potential of these agents as a stroke therapy.

## Background

Minimising brain injury following stroke is a critical clinical goal both to improve patient quality of life and to lessen the social and economic impacts of this devastating disorder. Currently, the most effective stroke therapy is to restore cerebral blood flow to a blocked artery using tPA and thrombectomy [1–3]. However, the current therapeutic window for coupled tPA ± thrombectomy therapy is so narrow (4.5 h) that the majority of stroke patients are unable to receive the treatment. Moreover, for those that do, up to 7 % develop intracranial haemorrhage as a complication. In addition, tPA ± thrombectomy is only available to patients having ready access to a hospital that has the facilities required for performing the procedures. Other treatments are only suitable for a small proportion of patients (e.g. hemicraniectomy to reduce intracranial pressure due to cerebral oedema) or provide only modest benefit (e.g. aspirin to reduce risk of clot propagation) [4]. As a consequence, while recent improvements in stroke therapy have been made, these have been limited and it is clear that there is urgent need for new, more widely applicable neuroprotective therapies that can be applied to stroke patients early by ambulance paramedics, in hospital emergency departments, and in remote locations away from tertiary hospitals. Additionally, any treatment that might improve the safety, therapeutic window and neuroprotective outcomes for tPA ± thrombectomy would be of great clinical significance.

Against the backdrop of the limited nature of current therapies, we have recently demonstrated that poly-arginine (and arginine-rich) peptides have potent neuroprotective properties in in vitro injury models that mimic the effects of stroke [5–7]. We have also established that poly-arginine peptides, as well as other arginine-rich peptides, including TAT and penetratin belonging to a class of peptide with cell penetrating properties also possess intrinsic neuroprotective properties [5–7]. Moreover, our in vitro data show that neuroprotective potency is enhanced with increasing arginine content (e.g. polymer length) [6]. As evidence of their clinical applicability, we have demonstrated that the poly-arginine R9D significantly reduces infarct volume in vivo following permanent middle cerebral artery occlusion (MCAO) in the rat [6]. A recent report [8] has also demonstrated that poly-arginine 7 (R7) containing peptides are neuroprotective in an in vivo retinal ganglion NMDA excitotoxicity model.

The neuroprotective properties of poly-arginine peptides in vitro and in vivo suggest that they may have potential as a neuroprotective therapy for stroke patients. To further investigate the efficacy of poly-arginine peptides in vivo and given the positive results obtained with the R9D peptide, in this study we assess the neuroprotective efficacy of the longer L-isoform poly-arginine peptides R12, R15 and R18 when administered 30 min after permanent MCAO. In addition, unlike in our earlier R9D trial, this study assesses functional outcomes using three behavioural tests as well as infarct volume to gain an understanding of the functional consequences of neuroprotection.

## Results

### Physiological and infarct volume measurements

**Fig. 1:**
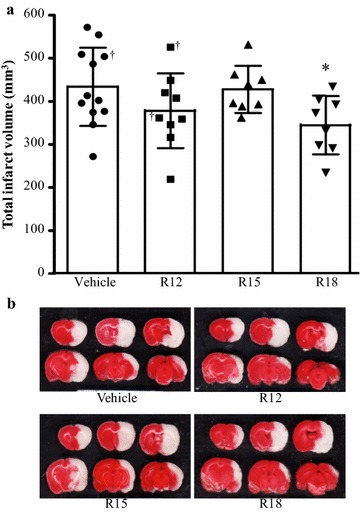
Infarct volume measurements and coronal brain slices 24 h after permanent MCAO. Treatments were administered intravenously (saline vehicle or peptide 1000 nmol/kg; in 600 µl volume over 6 min) 30 min after MCAO. **a** Values are mean ± SD. *P < 0.05 when compared to the vehicle control group. ^†^Denotes animals that died the following day after surgery, before the 24 h post-MCAO end-point, but whose infarct volume was measured nonetheless. **b** Representative TTC coronal brain slices from vehicle and peptide treated animals

**Fig. 2:**
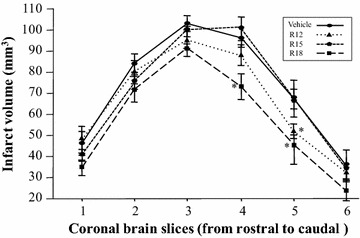
Infarct volume analysis in coronal brain slices (1–6 from rostral to caudal). The R18 peptide significantly reduced injury in brain slices 4 and 5, and the R12 peptide significantly reduced brain injury in slice 5. Values are mean ± SD; *P < 0.05 when compared to the vehicle control group

Physiological measurements before or during surgery confirmed the absence of any significant differences between animal treatment groups (Table 1). Data on the mean total infarct volumes and representative TTC stained coronal brain slices for each treatment group are presented in Fig. 1. These results show that the R18 peptide significantly reduced infarct volume (20.5 % reduction; *P* = 0.014). The R12 peptide also reduced infarct volume (12.8 % reduction), but not to a statistical significant extent (*P* = 0.105). By contrast, the R15 peptide had no effect on infarct volume. Rostral to caudal topographic analysis of infarcts in brain slices revealed that the R18 peptide significantly reduced brain injury in coronal slices 4 (*P* = 0.008) and 5 (*P* = 0.01) (Fig. 2). In addition, the R12 peptide significantly reduced brain injury in coronal slice 5 (*P* = 0.027).

**Table 1:** Physiological parameters for experimental animals used in study

	Saline (N = 12)	R12 (N = 9)	R15 (N = 8)	R18 (N = 8)
PaO_2_ (mmHg)	115.10 ± 33.51	124.30 ± 18.40	112.80 ± 16.96	120.60 ± 20.34
PaCO_2_ (mmHg)	42.92 ± 5.82	46.00 ± 4.21	39.38 ± 5.20	44.25 ± 7.74
pH	7.44 ± 0.09	7.33 ± 0.08	7.31 ± 0.09	7.42 ± 0.08
Glucose (mmol/L)	7.74 ± 1.27	7.42 ± 1.06	7.03 ± 1.11	7.13 ± 1.08
Blood pressure (mmHg)	89.00 ± 8.44	78.44 ± 6.98	88.00 ± 9.97	79.63 ± 12.65
Body temperature (°C)	37.48 ± 0.18	37.58 ± 0.13	37.46 ± 0.21	37.51 ± 0.06

PaO_2_, PaCO_2_, pH, blood pressure and glucose measured before MCAO. Body temperature data represent average over 2 h post-surgery monitoring period. Data are mean ± SD

There were three post-treatment animal deaths that occurred the day following surgery, one in the vehicle and two in the R12-treated animals. While the animal deaths could be directly related to stroke severity and/or treatment, the exact cause of the deaths could not be precisely determined on autopsy.

### Functional outcome assessment

**Fig. 3:**
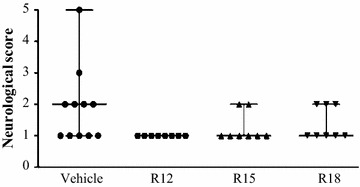
Neurological grading scores 24 h after permanent MCAO (0 = no deficit, 4 = major deficit) for saline (vehicle) and peptide (R12, R15, R18; 1000 nmol/kg) treatment groups. Assessment was performed immediately before euthanasia. Lines on graph indicate range and median for neurological scores

**Fig. 4:**
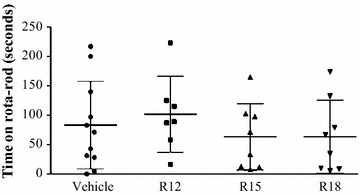
Rota-rod performance 24 h after permanent MCAO for saline (vehicle) and peptide (R12, R15, R18; 1000 nmol/kg) treatment groups. Results for this test were highly variable within groups and no significant differences were detected. Average time healthy pre-surgery animals remained on rota-rod was 78 s (data not shown). Values are mean ± SD

Neurological scores using the modified Bederson’ scale for each treatment group are presented in Fig. 3. While neurological scores did not differ statistically between groups, the vehicle control group score was higher (1.9) than any of the scores for the peptide treatment groups (<1.4), indicative of a possible positive treatment effect. Results for the rota-rod assessment for each treatment group are presented in Fig. 4. Results were highly variable within groups and no significant differences were detected.

**Fig. 5:**
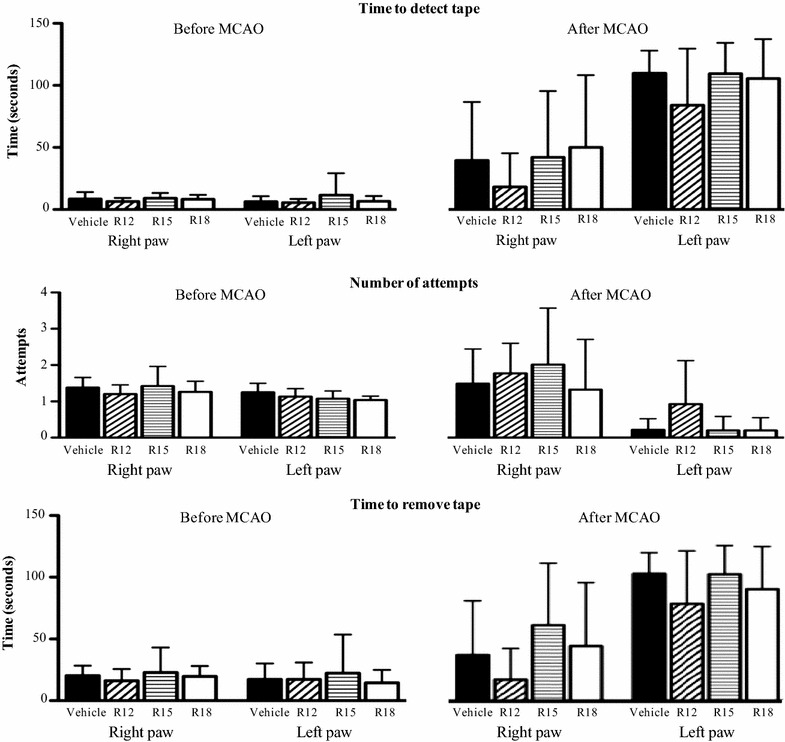
Functional assessment measurements using adhesive tape removal test before and 24 h after MCAO for saline (vehicle) and peptide (R12, R15, R18; 1000 nmol/kg) treatment groups. Post-MCAO assessment was performed immediately before euthanasia. No treatment significantly improved adhesive tape detection or removal times for the *left* or *right paw*. Values are mean ± SD; N = 11 for vehicle, N = 7 for R12, N = 8 for R15 and N = 8 for R18. Maximum time allowed for adhesive tape removal was 120 s

For the adhesive tape removal test pre- and post-MCAO measurements for time to detect tape, the number of attempts to remove tape and time taken to remove tape for each treatment group are presented in Fig. 5. As expected, the left paw was more adversely affected than the right paw, however there were no statistically significant differences between vehicle-treated versus peptide-treated groups. However, for the R12 peptide all parameters measured for the left paw, and two out of the three measurements obtained for the right paw showed a positive improvement, albeit not to a statistically significant extent.

### Weight loss measurement

**Fig. 6:**
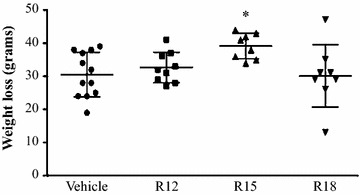
Weight loss at 24 h after permanent MCAO for saline (vehicle) and peptide (R12, R15, R18; 1000 nmol/kg) treatment groups. Values are mean ± SD; *P < 0.05 when compared to the vehicle control group

At experiment end, all treatment groups recorded a loss in weight, with the greatest weight loss occurring in the R15 peptide treatment group (*P* = 0.004; Fig. 6).

## Discussion

In a previous study, we demonstrated that the poly-arginine peptide R9D could reduce infarct volume by 20 % when administered intravenously 30 min post-MCAO [6], however no functional assessment was performed. The present study extends this previous study to include the poly-arginine peptides R12, R15 and R18 and explores their capacity to reduce infarct volume and improve functional outcomes when administered intravenously 30 min post-MCAO. Whereas R15 had no effect on infarct volume, R18 significantly reduced infarct volume (20.5 % reduction) and there was a trend towards reduced infarct volume with R12 (12.8 % reduction). Importantly, all peptide treatments displayed a trend towards improvement in one or more of the neurological functional tests. Whilst the level of infarct volume reduction was modest (12.8–20.5 %), this most likely reflects the severity of the stroke model used in this particular study where up to 90 % of the affected brain hemisphere is infarcted by the stroke. It is also likely that the modest reductions in infarct volume, stroke severity and 24-h endpoint coupled with the small animal numbers used explain why the trend towards improvements in functional outcomes was not statistically significant. Despite the modest effects of the poly-arginine peptides following permanent MCAO, it is still possible that these peptides have potential clinical application, especially in less severe forms of stroke, stroke associated with cerebral reperfusion treatments (tPA ± thrombectomy) and haemorrhagic stroke.

With respect to neuroprotective efficacy, further research is required to determine the optimal dose of the peptides to reduce infarct volume. It was particularly surprising that the R15 peptide did not have any affect on infarct volume reduction, despite showing comparable neuroprotective efficacy to R18 when assessed in an in vitro neuronal glutamate excitotoxicity model [6]. The reason why no observable neuroprotection was obtained for R15 is at present unknown, but it is possible that a higher or lower dose may be more effective than the dose used in the current study. Studies are currently underway in our laboratory to more definitively address questions surrounding effective dosage for a range of poly-arginine peptides in the in vivo stroke model.

The present study did not investigate the mechanism of action of peptides, but in previous studies we have shown that poly-arginine peptides have the capacity to reduce excitotoxic glutamic acid-induced calcium influx in cultured cortical neurons [6, 7]. Based on this finding, as well as the findings of other studies, we have hypothesised that these peptides have the capacity to inhibit calcium influx by causing the internalisation of cell surface structures such as ion channels and thereby reduce the toxic neuronal calcium entry that occurs after excitotoxicity and cerebral ischemia. We have speculated that due to the cell penetrating properties of arginine-rich peptides, including putative “neuroprotective peptides” fused to the arginine-rich carrier peptide TAT, ion channel receptor internalisation occurs during neuronal endocytic uptake of the peptides [6, 7]. Evidence that supports our hypothesis includes studies demonstrating that arginine-rich peptides: (1) interfere with the function of NMDA [9–14] and vanilloid receptors [15], voltage gated calcium channels [16–18] and the sodium calcium exchanger [13]; (2) cause internalisation or reduced surface expression of neuronal ion channels [11, 13, 18]; and (3) can induce the endocytic internalisation of epidermal growth factor receptor and tumour necrosis factor receptors in HeLa cells [19].

In support of the poly-arginine neuroprotective findings in the present study, a recent report [8] has confirmed the neuroprotective properties of poly-arginine 7 (R7) containing peptides and other arginine-rich peptides (TAT and TATNR2B9c) in an in vivo retinal ganglion NMDA excitotoxicity model. Moreover, the study also provides evidence for an additional neuroprotective mechanism associated with maintenance of mitochondrial function and integrity.

Studies in our laboratory to confirm peptide-induced internalisation of cell surface receptors and other neuroprotective mechanisms are in progress. While we have demonstrated that arginine-rich peptides have the capacity to reduce excitotoxic calcium influx, it will be important to obtain a more comprehensive understanding of peptide neuroprotective mechanism of action. Nevertheless our findings indicate poly-arginine peptides have both in vitro and in vivo neuroprotective properties and warrant further evaluation in different stroke models and other acute brain injury disorders.

## Conclusion

The findings of this study further validates the neuroprotective properties of poly-arginine peptides [5–9], highlights their status a new class of neuroprotective agent and provides justification for their evaluation in different stroke models and other acute brain injury disorders. The findings also further question the mechanism of action of the many reported “neuroprotective peptides” fused to arginine-rich carrier peptides, which are thought to act through interaction with specific intracellular proteins, but which our data suggest may act through a common mechanism of action relating to peptide arginine content and positive charge.

## Methods

### Peptides

The R12 (H-RRRRRRRRRRRR-OH), R15 (H-RRRRRRRRRRRRRRR-OH) and R18 H-RRRRRRRRRRRRRRRRRR-OH) peptides used in the study were synthesised by China Peptides (Shanghai, China). The peptides were HPLC purified to >94 % purity. All peptides were prepared in 0.9 % sodium chloride for injection (Pfizer, Perth, Australia) aliquoted into 650 µl volumes in 3 ml syringes and stored at −20 °C until use.

### Rat permanent middle cerebral artery occlusion procedure

This study was approved by the Animal Ethics Committee of the University of Western Australia and follows guidelines outlined by the *Australian Code for the Care and use of Animals for Scientific Purposes*. The experimental procedure for performing the permanent middle cerebral artery occlusion (MCAO) stroke model has been described previously [20, 21]. Briefly, male Sprague–Dawley rats weighing 270–320 g were kept under controlled housing conditions with a 12 h light–dark cycle and with free access to food and water. Experimental animals were fasted overnight and subjected to filament permanent MCAO. In order to monitor blood pressure and withdraw blood samples, a cannula was inserted in the tail artery. Between 50 and 200 µL of blood was used for glucose (glucometer; MediSense Products, Abbott Laboratories, Bedford, MA, USA) and other measurements (PaO_2_, PaCO_2_, pH; ABL5, Radiometer, Copenhagen, Denmark). The MCAO procedure was considered successful based on a >25 % decrease from baseline in cerebral blood flow (CBF) after insertion of filament, as measured by laser Doppler flowmetry. During surgery temperature was closely monitored using a rectal probe (Physitemp Instruments, Clifton, USA) and maintained at 37.5 ± 0.5 °C, with fan heating or cooling.

Thirty minutes post-MCAO, rats were intravenously treated with the peptide (1000 nmol/kg in 600 µL over 6 min) or vehicle (0.9 % sodium chloride for injection; 600 µL over 6 min). Treatments were administered via the right internal jugular vein and infusion pump. Treatments were randomised and all procedures were performed blinded to treatment.

Twenty-fours hours post-MCAO, infarct area assessment was performed by preparing 2 mm thick cerebral coronal brain slices, and incubating in 3 % 2,3,5 triphenyltetrazolium chloride (TTC; Sigma-Aldrich, St. Louis, USA) at 37 °C for 20 min, followed by fixation in 4 % formalin at room temperature overnight. Digital images of coronal sections were acquired using a colour scanner and analysed by an operator blind to treatment status, using ImageJ software (3rd edition, NIH, Bethesda, USA). The total infarct volume was determined by measuring the areas of infarcted tissue on both sides of the 2 mm sections. These measured areas were corrected for cerebral oedema by multiplying the infarct volume for the oedema index (calculated by dividing the total volume of the stroke-affected hemisphere by the total volume of the contralateral hemisphere) [22].

A total of 42 animals were used in the trial. Five animals were excluded from the study; two animals were euthanased due to subarachnoid haemorrhage, one animal was excluded due to insufficient decrease in CBF, one animal was excluded due to pyrexia, and one died during surgical recovery for an unknown reason.

### Post-surgical monitoring

Following surgery animals were placed in a clean cage with free access to food and water. The body temperature of animals was measured every 30–60 min using a rectal probe for at least 2 h post-surgery, and maintained between 37.0 and 37.8 °C. To avoid hypothermia, rat cages were placed on a heating mat during the post-surgical monitoring and housed in a holding room maintained at 26–28 °C. If necessary, additional heating or cooling was performed by applying fan heating or cold water spray.

### Behavioural testing

To determine if peptide treatment was associated with improved sensorimotor outcomes, three neurological tests were performed 24 h post-stroke.

### Neurological assessment test

The scoring system was performed using the modified Bederson’ scale. Scores range from 0 for no deficits, 1 for flexed forepaw, 2 for inability to resist lateral push, 3 for circling, 4 for agitated circling and 5 for unresponsive to stimulation/stupor [23].

### Adhesive tape removal test

This is a bilateral asymmetry paw-test, which assesses sensorimotor impairment [24]. Adhesive tape (Diversified Biotech, Dedham, USA) 10 mm × 10 mm in size was placed on the palmar surface of the forepaw and the time taken for the first attempt to remove tape, the number of attempts to remove tape and the total time taken to remove tape recorded. Each forelimb was assessed sequentially starting with the unaffected side (right side) with animals having a maximum of 120 s to complete the task (normal rats usually take between 5 and 30 s to remove the tape). Animals were tested a total of six times, three times on the day before surgery and three times 24 h post-MCAO. Mean values were calculated for each forepaw for the pre- and post-surgery trials.

### Rota-rod test

This test assesses balance and coordination by assessing a rat’s ability to remain walking on a rotating rod when its speed of rotation gradually increases from 4 to 40 revolutions per minute. The time at which the animal falls is recorded. Typically rats fall 27–137 s after placement on the rod.

### Statistical analysis

Mean infarct volume measurements (total and coronal slices) for each treatment group was compared to the vehicle control group by analysis of variance (ANOVA) followed by the Fisher’s post hoc analysis. Data from neurological assessment were analysed using Kruskal–Wallis test [25]. Data from adhesive tape and rota-rod tests were analysed using ANOVA followed by post hoc analysis using Scheffe’s multiple comparison procedure. A value of *P* < 0.05 was considered significant for all data sets. Data in figures are presented as mean ± standard deviation (SD).
